# A Unified Model for Voltage-Source and Current-Source Virtual Synchronous Generators

**DOI:** 10.3390/e28101061

**Published:** 2026-09-26

**Authors:** Dongdong Wang, Xiongfeng Luo, Haiwei Geng, Xia Yu, Yinbing Yang, Yilin Tang

**Affiliations:** 1 School of Electrical and Information Engineering, Hunan Institute of Technology, Hengyang 421002, China; yuxia@hnit.edu.cn (X.Y.); 19173181447@163.com (Y.Y.); tyl867176794@163.com (Y.T.); 2 Hengyang City Metrology Testing Center, Hengyang 421002, China; luoxiongfenguniv@163.com (X.L.); 18073496123@163.com (H.G.)

**Keywords:** virtual synchronous generator, virtual permanent-magnet synchronous generator, virtual permanent-capacitor synchronous generator, voltage-source inverter, current-source inverter, stability analysis

## Abstract

The virtual synchronous generator serves as a critical interface between renewable generation systems and the grid, providing inertia to support both active and reactive power. Based on the duality principle, a virtual permanent-capacitor synchronous generator is derived to enhance the inertia of the three-phase current-source inverter, exhibiting dynamic characteristics similar to those of a virtual permanent-magnet synchronous generator. Additionally, this work presents a unified virtual synchronous generator model applicable to both voltage-source and current-source topologies. By constructing a suitable Lyapunov function candidate, the stability criteria of the unified model under standalone operation are established. The Lyapunov function candidate is constructed as a Bregman divergence, whose monotonic decrease characterizes the convergence to synchronous equilibrium. In grid-connected mode, a frequency-locking condition is derived to ensure synchronization of the virtual synchronous generators with the grid. Eigenvalue analysis is employed to assess the stability of the unified model under frequency-locking conditions. The correctness of the derived stability conditions in standalone and grid-connected modes is confirmed through time-domain simulations.

## 1. Introduction

Virtual synchronous generators (VSGs) are widely adopted in renewable generation systems to provide virtual inertia for supporting grid voltage and frequency [1]. Two primary control strategies are derived from the PQ dynamics and synchronous generator dynamic equations [1,2,3,4,5]. The former employs second-order PQ dynamics to regulate active and reactive power for grid frequency and voltage stabilization; however, it cannot fully replicate the dynamic characteristics of synchronous generators. In contrast, the latter serves as a prominent inertia emulation approach that comprehensively captures flux linkage dynamics, the swing equation, and additional electromechanical transients.

VSGs are classified into voltage-controlled and current-controlled types, which function as voltage sources and current sources, respectively [6]. Voltage-controlled VSGs are better suited to weak grids, whereas current-controlled VSGs perform better in strong grids. Conventional voltage-controlled VSGs are based on the mathematical model of synchronous generators, incorporating stator voltage dynamics, flux linkage equations, and mechanical torque relationships [7,8,9,10].

To further simplify the control strategy, a permanent-magnet synchronous generator (PMSG) model can be adopted for VSG implementation, requiring fewer equations while preserving essential dynamic characteristics [11]. However, under strong grid conditions, voltage-source inverter (VSI)-based VSGs may induce instability. To guarantee safe grid integration, a comprehensive stability analysis of the VSI must be conducted [12]. Although current-controlled VSGs have been proposed to mitigate instability, they remain fundamentally dependent on VSIs, whose intrinsic voltage-source characteristics limit current regulation fidelity [13,14,15]. Moreover, the phase-locked loop (PLL), an essential component of conventional voltage-controlled VSGs, can itself become a source of instability [16,17].

A duality exists between grid-forming and grid-following inverters [18]. More broadly, this concept can be generalized to the relationship between voltage-source inverters (VSIs) and current-source inverters (CSIs) illustrated in Figure 1 [19,20,21]. Exploiting the duality between the averaged VSI and CSI models [22], this paper proposes a CSI-based VSG—the dual counterpart of the virtual permanent-magnet synchronous generator (VPMSG)—to achieve inertia control for CSI systems. Accordingly, leveraging the three-phase CSI topology shown in Figure 1b, this paper proposes the virtual permanent-capacitor synchronous generator (VPCSG) in Section 2. The duality between the VPMSG and the VPCSG is further elaborated. Section 3 establishes a unified mathematical model that encompasses both the VPMSG and the VPCSG. In Section 4, the stability condition for the unified model in standalone mode is determined via a Lyapunov function candidate, and eigenvalue analysis is performed to assess its grid-connected stability. In this work, we recognize *V* as a Bregman divergence $D_{\phi }(\tilde{z},0)$ with the convex generator $\phi (\tilde{z})={\tilde{z}}^{T}P\tilde{z}$, whose monotonic decrease verifies the stability of the VSGs. Subsequently, Section 5 validates the dynamic stability of both the VPMSG and the VPCSG through time-domain simulations.

## 2. Derivation of the Virtual Permanent-Capacitor Synchronous Generator Model

Following the space vector formulation of PMSG stator voltage equations in [23,24,25], the VPMSG counterpart is derived analogously as

(1) $${\vec{v}}_{s}=-R_{s}{\vec{i}}_{s}+\frac{d{\vec{\varphi }}_{s}}{dt}$$

Here, the virtual stator voltage, current, and flux vectors are defined as

(2) $${\vec{v}}_{s}=\frac{2}{3}(v_{a}+v_{b}e^{j\frac{2\pi }{3}}+v_{c}e^{j\frac{4\pi }{3}})$$

(3) $${\vec{i}}_{s}=\frac{2}{3}(i_{a}+i_{b}e^{j\frac{2\pi }{3}}+i_{c}e^{j\frac{4\pi }{3}})$$

(4) $${\vec{\varphi }}_{s}=-L_{s}{\vec{i}}_{s}+\psi_{r}e^{j\theta_{e}}$$

where *v*_a_, *v*_b_, and *v*_c_ are the virtual phase voltages; *i*_a_, *i*_b_, and *i*_c_ are the virtual phase currents; and *L*_s_, *ψ*_r_, and *θ*_e_ are the virtual stator inductance, the virtual flux amplitude, and the virtual electrical angle of the rotor, respectively. *θ*_e_ = *ω*_e_*t*, where *ω*_e_ is the virtual electrical angular frequency of the rotor. *ω*_e_ = *n*_p_*ω*_m_, where *ω*_m_ and *n*_p_ are the virtual mechanical angular frequency and the virtual number of pole pairs, respectively. By the duality principle [26], dual circuits share identical mathematical structures; thus, the dual of Equation (1) is expressed as

(5) $${\vec{\hat{i}}}_{s}=-{\hat{G}}_{s}{\vec{\hat{v}}}_{s}+\frac{d{\vec{\hat{q}}}_{s}}{dt}$$

The duality correspondence between Equations (1) and (5) is summarized in Table 1.

Here, the dual counterparts of the virtual stator voltage, current, and flux vectors—namely, the current, voltage, and charge vectors—are defined as follows:

(6) $${\vec{\hat{i}}}_{s}=\frac{2}{3}({\hat{i}}_{a}+{\hat{i}}_{b}e^{j\frac{2\pi }{3}}+{\hat{i}}_{c}e^{j\frac{4\pi }{3}})$$

(7) $${\vec{\hat{v}}}_{s}=\frac{2}{3}({\hat{v}}_{a}+{\hat{v}}_{b}e^{j\frac{2\pi }{3}}+{\hat{v}}_{c}e^{j\frac{4\pi }{3}})$$

(8) $${\vec{\hat{q}}}_{s}=-{\hat{C}}_{s}{\vec{\hat{v}}}_{s}+{\hat{q}}_{r}e^{j{\hat{\theta }}_{e}}$$

Henceforth, the hat symbol (ˆ) denotes dual quantities. ${\hat{i}}_{a}$, ${\hat{i}}_{b}$, and ${\hat{i}}_{c}$ are the dual phase currents; ${\hat{v}}_{a}$, ${\hat{v}}_{b}$, and ${\hat{v}}_{c}$ are the dual phase voltages; ${\hat{C}}_{s}$, ${\hat{q}}_{r}$, and ${\hat{\theta }}_{e}$ are the dual capacitance, the dual charge amplitude, and the dual electrical angle, respectively. ${\hat{\theta }}_{e}={\hat{\omega }}_{e}t$, where ${\hat{\omega }}_{e}$ denotes the dual electrical angular frequency. Projecting the *abc*-frame space vectors onto the *dq* frame yields the *dq*-axis current and voltage components, namely,

(9) $${{\vec{\hat{i}}}_{s}}^{\prime }={\vec{\hat{i}}}_{s}e^{-j{\hat{\theta }}_{e}}={\hat{i}}_{d}+j{\hat{i}}_{q}$$

(10) $${{\vec{\hat{v}}}_{s}}^{\prime }={\vec{\hat{v}}}_{s}e^{-j{\hat{\theta }}_{e}}={\hat{v}}_{d}+j{\hat{v}}_{q}$$

Here, ${\hat{i}}_{d}$ and ${\hat{i}}_{q}$ are the *dq*-axis components of the dual current vector, and ${\hat{v}}_{d}$ and ${\hat{v}}_{q}$ are the *dq*-axis components of the dual voltage vector.

Substituting Equation (8) into Equation (5) yields

(11) $${\vec{\hat{i}}}_{s}=-{\hat{G}}_{s}{\vec{\hat{v}}}_{s}-{\hat{C}}_{s}\frac{d{\vec{\hat{v}}}_{s}}{dt}+j{\hat{\omega }}_{e}{\hat{q}}_{r}e^{j{\hat{\theta }}_{e}}$$

Multiplying Equation (11) by $e^{-j{\hat{\theta }}_{e}}$ yields

(12) $${\vec{\hat{i}}}_{s}e^{-j{\hat{\theta }}_{e}}=-{\hat{G}}_{s}{\vec{\hat{v}}}_{s}e^{-j{\hat{\theta }}_{e}}-{\hat{C}}_{s}\frac{d{\vec{\hat{v}}}_{s}}{dt}e^{-j{\hat{\theta }}_{e}}+j{\hat{\omega }}_{e}{\hat{q}}_{r}$$

Differentiating Equation (10) with respect to *t* yields

(13) $$\frac{d{\vec{\hat{v}}}_{s}}{dt}e^{-j{\hat{\theta }}_{e}}=\frac{d{{\vec{\hat{v}}}_{s}}^{\prime }}{dt}+j{\hat{\omega }}_{e}{{\vec{\hat{v}}}_{s}}^{\prime }$$

Substituting Equation (13) into Equation (12) yields

(14) $${{\vec{\hat{i}}}_{s}}^{\prime }=-{\hat{G}}_{s}{{\vec{\hat{v}}}_{s}}^{\prime }-{\hat{C}}_{s}\frac{d{{\vec{\hat{v}}}_{s}}^{\prime }}{dt}-{\hat{C}}_{s}j{\hat{\omega }}_{e}{{\vec{\hat{v}}}_{s}}^{\prime }+j{\hat{\omega }}_{e}{\hat{q}}_{r}$$

Equations (9) and (10) are substituted into Equation (14), which yields

(15) $$\left\{\begin{array}{l} {\hat{C}}_{s}\frac{d{\hat{v}}_{d}}{dt}=-{\hat{i}}_{d}-{\hat{G}}_{s}{\hat{v}}_{d}+{\hat{\omega }}_{e}{\hat{C}}_{s}{\hat{v}}_{q}\\ {\hat{C}}_{s}\frac{d{\hat{v}}_{q}}{dt}=-{\hat{i}}_{q}-{\hat{G}}_{s}{\hat{v}}_{q}-{\hat{\omega }}_{e}{\hat{C}}_{s}{\hat{v}}_{d}+{\hat{q}}_{r}{\hat{\omega }}_{e} \end{array}\right.$$

The dual torque equation for the VPMSG is given by

(16) $$J\frac{d{\hat{\omega }}_{e}}{dt}={\hat{n}}_{p}T_{m}-{\hat{n}}_{p}{\hat{T}}_{e}-B_{f}{\hat{\omega }}_{e}$$

Here, *J*, ${\hat{T}}_{e}$, *T*_m_, and *B*_f_ are the virtual total inertia, the virtual electromagnetic torque, the virtual mechanical torque, and the virtual viscous friction coefficient, respectively; ${\hat{\omega }}_{e}={\hat{n}}_{p}{\hat{\omega }}_{m}$, where ${\hat{\omega }}_{m}$ and ${\hat{n}}_{p}$ are the dual mechanical angular frequency and the dual virtual number of pole pairs.

Multiplying Equation (8) by $e^{-j{\hat{\theta }}_{e}}$ yields

(17) $${{\vec{\hat{q}}}_{s}}^{\prime }={\vec{\hat{q}}}_{s}e^{-j{\hat{\theta }}_{e}}=-{\hat{C}}_{s}({\hat{v}}_{d}+j{\hat{v}}_{q})+{\hat{q}}_{r}={\hat{q}}_{d}+j{\hat{q}}_{q}$$

Thus,

(18) $$\left\{\begin{array}{l} {\hat{q}}_{d}=-{\hat{C}}_{s}{\hat{v}}_{d}+{\hat{q}}_{r}\\ {\hat{q}}_{q}=-{\hat{C}}_{s}{\hat{v}}_{q} \end{array}\right.$$

The output electrical power is given by

(19) $$\begin{array}{l} {\hat{p}}_{s}=\frac{3}{2}({\hat{i}}_{d}{\hat{v}}_{d}+{\hat{i}}_{q}{\hat{v}}_{q})\\ =\frac{3}{2}{\hat{q}}_{r}{\hat{\omega }}_{e}{\hat{v}}_{q}-\frac{3}{2}({\hat{C}}_{s}\frac{d{\hat{v}}_{d}}{dt}{\hat{v}}_{d}+{\hat{C}}_{s}\frac{d{\hat{v}}_{q}}{dt}{\hat{v}}_{q})-\frac{3}{2}({\hat{G}}_{s}{{\hat{v}}_{d}}^{2}+{\hat{G}}_{s}{{\hat{v}}_{q}}^{2})\\ ={\hat{p}}_{\mathrm{em}}-\frac{3}{2}({\hat{C}}_{s}\frac{d{\hat{v}}_{d}}{dt}{\hat{v}}_{d}+{\hat{C}}_{s}\frac{d{\hat{v}}_{q}}{dt}{\hat{v}}_{q})-{\hat{p}}_{\mathrm{loss}} \end{array}$$

In Equation (19), the first term represents the virtual input mechanical power, the second term denotes the capacitive power stored in the electric field, and the third term accounts for the power losses. The virtual input mechanical power is defined as

(20) $${\hat{p}}_{\mathrm{em}}={\hat{T}}_{e}{\hat{\omega }}_{m}$$

Thus, the virtual electromagnetic torque is defined as

(21) $${\hat{T}}_{e}=\frac{3}{2}{\hat{n}}_{p}({\hat{q}}_{d}{\hat{v}}_{q}-{\hat{q}}_{q}{\hat{v}}_{d})=\frac{3}{2}{\hat{n}}_{p}{\hat{q}}_{r}{\hat{v}}_{q}$$

Equations (15), (16), and (21) form the VPCSG model, which has a mathematical structure similar to that of the VPMSG. Thus, the VPCSG replicates the dominant dynamic characteristics of the VPMSG when the dual operating parameters are set to identical values. Finally, the duality mappings between the VPMSG and VPCSG are summarized in Table 2. Depending on the circuit topology, the swing equations listed in Table 2 are either emulated through virtual inertia control or physically realized via the actual electromechanical dynamics of the circuit. However, implementing the virtual swing equation increases control complexity due to additional state variables and tuning parameters, whereas the physical electromechanical swing equation inherently simplifies controller design by leveraging natural system dynamics. For the topologies shown in Figure 1, the dc-link capacitor equation of the VSI and the dc-link inductor equation of the CSI serve as the respective swing equations. Therefore, explicit implementation of the swing equation in the control strategy becomes unnecessary.

Thus, the duality between VPMSG and VPCSG for the topologies in Figure 1 is summarized in Table 3. Here, *i*_dc_ and *v*_dc_ denote the input current and capacitor voltage of the VSI, respectively; ${\hat{i}}_{L}$ and ${\hat{v}}_{\mathrm{dc}}$ denote the inductor current and input voltage of the CSI, respectively. The electrical angular frequency is given by *ω*_e_ = *n*_p_*v*_dc_ for the VPMSG, while for the dual VPCSG, it follows ${\hat{\omega }}_{e}={\hat{n}}_{p}{\hat{i}}_{L}$. The proportionality constant bridging the dc-link quantity (${\hat{i}}_{L}$ for VPCSG, or *v*_dc_ for VPMSG) and the electrical angular frequency has units of [rad/(s·A)] in the VPCSG case (or [rad/(s·V)] in the VPMSG case). The virtual number of pole pairs in ${\hat{\omega }}_{e}={\hat{n}}_{p}{\hat{i}}_{L}$ for CSI (or *ω*_e_ = *n*_p_*v*_dc_ for VSI) absorbs the necessary scaling so that

- ${\hat{n}}_{p}$ has units of rad/(s·A) for VPCSG;
- *n*_p_ has units of rad/(s·V) for VPMSG.

In the proposed VPMSG/VPCSG framework, the virtual number of pole pairs functions mathematically as a virtual scaling gain rather than a physical pole-pair count, since there are no actual magnetic poles. We adopted the term “number of pole pairs” to maintain analogy with the conventional synchronous machine model from which the swing equation is borrowed. In the VPCSG, ${\hat{T}}_{e}$ is a virtual torque that is mathematically dual to the virtual electromagnetic torque in the VPMSG. It does not correspond to a physical electromagnetic torque (there is no magnetic field or rotor). Instead, it is a power-derived control quantity. Physically, ${\hat{T}}_{e}$ in the VPCSG denotes ${\hat{v}}_{\mathrm{dc}}$.

To derive control strategies for the VPMSG and the VPCSG, the dynamic equations of the VSI and CSI in Figure 1 are formulated as follows:

(22) $$\mathrm{VSI}:\;\left\{\begin{array}{l} L_{s}\frac{di_{d}}{dt}=-v_{d}-R_{s}i_{d}+\omega_{e}L_{s}i_{q}+u_{d}\\ L_{s}\frac{di_{q}}{dt}=-v_{q}-R_{s}i_{q}-\omega_{e}L_{s}i_{d}+u_{q}\\ C\frac{dv_{\mathrm{dc}}}{dt}=I_{s}-i_{\mathrm{dc}}-Gv_{\mathrm{dc}} \end{array}\right.$$

(23) $$\mathrm{CSI}:\;\left\{\begin{array}{l} {\hat{C}}_{s}\frac{d{\hat{v}}_{d}}{dt}=-{\hat{i}}_{d}-{\hat{G}}_{s}{\hat{v}}_{d}+{\hat{\omega }}_{e}{\hat{C}}_{s}{\hat{v}}_{q}+{\hat{i}}_{\mathrm{id}}\\ {\hat{C}}_{s}\frac{d{\hat{v}}_{q}}{dt}=-{\hat{i}}_{q}-{\hat{G}}_{s}{\hat{v}}_{q}-{\hat{\omega }}_{e}{\hat{C}}_{s}{\hat{v}}_{d}+{\hat{i}}_{\mathrm{iq}}\\ \hat{L}\frac{d{\hat{i}}_{L}}{dt}={\hat{V}}_{s}-{\hat{v}}_{\mathrm{dc}}-\hat{R}{\hat{i}}_{L} \end{array}\right.$$

Here, *u*_d_ and *u*_q_ are the *dq*-axis components of the output voltages of the VSI in Figure 1a; ${\hat{i}}_{\mathrm{id}}$ and ${\hat{i}}_{\mathrm{iq}}$ are the *dq*-axis output current components of the CSI in Figure 1b. The conductance value in the model is an equivalent parallel conductance derived from the capacitor’s equivalent series resistance (ESR).

The control references for the VPMSG are obtained by mapping the VSI dynamic equations onto the VPMSG model, which directly yields the voltage reference expressions for the VSI. A similar derivation applies to the VPCSG. The control strategies for the VPMSG and the VPCSG are summarized as follows:

VPMSG: *u*_d_* = 0 and *u*_q_* = *n*_p_*ψ*_r_*v*_dc_ (24)

(25) $$\mathrm{VPCSG}:\;{\hat{i}}_{\mathrm{id}}^{*}=0\;\mathrm{and}\;{\hat{i}}_{\mathrm{iq}}^{*}={\hat{n}}_{p}{\hat{q}}_{r}{\hat{i}}_{L}$$

Here, *u*_d_* and *u*_q_* represent the *dq*-axis reference voltages of the VSI; ${\hat{i}}_{\mathrm{id}}^{*}$ and ${\hat{i}}_{\mathrm{iq}}^{*}$ denote the *dq*-axis reference currents of the CSI; and *n*_p_, ${\hat{n}}_{p}$, *ψ*_r_, and ${\hat{q}}_{r}$ denote the control parameters. The control strategies for the VPMSG and VPCSG, given by Equations (24) and (25), are implemented via the SPWM modulation applied to the VSI and CSI in Figure 2. The proposed VSG features self-synchronization and is applicable to both standalone and grid-connected modes, thereby eliminating the need for a PLL in its control strategies. Given that the *dq*-axis reference voltages match the actual VSI output voltages, the VSI becomes equivalent to the VPMSG when Equation (24) is substituted into Equation (22); the VPCSG follows analogously from substituting Equation (25) into Equation (23). The corresponding *dq*-frame circuit equivalent models are derived as shown in Figure 3, confirming consistency with the models in Table 2 and Table 3. Here, $\theta_{e}=\int n_{p}v_{\mathrm{dc}}\mathrm{dt}$, ${\hat{\theta }}_{e}=\int {\hat{n}}_{p}{\hat{i}}_{L}\mathrm{dt}$. The references in Equations (24) and (25) are generated by sampling the dc-link quantity (*v*_dc_ for VPMSG, or ${\hat{i}}_{L}$ for VPCSG), in which *n*_p_, ${\hat{n}}_{p}$, *ψ*_r_, and ${\hat{q}}_{r}$ can be directly given (open-loop case) or obtained by the closed-loop controller. The open-loop configuration is adopted to derive a unified model for VSGs. These references are subsequently applied to a CSI modulator, subject to the following constraints: (1) satisfaction of the DC-link current continuity requirement and (2) avoidance of forbidden states that would cause an open circuit in the DC inductor. The modulation method used is SPWM adapted for CSI [27]. The three-phase current references are compared with a high-frequency triangular carrier to generate raw switching signals; the shorting pulse generator ensures that the dc-link inductor current is never interrupted and no destructive open circuit occurs.

However, circuit duality does not automatically imply dynamic equivalence; rather, it provides a structural correspondence between the two circuit topologies that, when combined with an appropriately dual control law, yields identical dynamics. Specifically, under the duality transformation in Table 1, the state equations of the VSI with L-filter and the CSI with C-filter map onto one another. When the VPMSG control is also transformed according to the same dual mapping, the averaged models of the VPMSG and the VPCSG become mathematically identical. The equivalence holds for the averaged dynamic model under both dual control design and circuit duality.

## 3. A Unified Modeling and Analysis Framework for Virtual Synchronous Generators

Based on Table 2 and Table 3, the VPMSG and VPCSG models are reformulated as follows:

(26) $$\mathrm{VPMSG}:\;\left\{\begin{array}{l} L_{s}\frac{di_{d}}{dt}=-v_{d}-R_{s}i_{d}+\omega_{e}L_{s}i_{q}\\ L_{s}\frac{di_{q}}{dt}=-v_{q}-R_{s}i_{q}-\omega_{e}L_{s}i_{d}+\psi_{r}\omega_{e}\\ C\frac{d\omega_{e}}{dt}=n_{p}I_{s}-\frac{3}{2}{n_{p}}^{2}\psi_{r}i_{q}-G\omega_{e} \end{array}\right.$$

(27) $$\mathrm{VPCSG}:\;\left\{\begin{array}{l} {\hat{C}}_{s}\frac{d{\hat{v}}_{d}}{dt}=-{\hat{i}}_{d}-{\hat{G}}_{s}{\hat{v}}_{d}+{\hat{\omega }}_{e}{\hat{C}}_{s}{\hat{v}}_{q}\\ {\hat{C}}_{s}\frac{d{\hat{v}}_{q}}{dt}=-{\hat{i}}_{q}-{\hat{G}}_{s}{\hat{v}}_{q}-{\hat{\omega }}_{e}{\hat{C}}_{s}{\hat{v}}_{d}+{\hat{q}}_{r}{\hat{\omega }}_{e}\\ \hat{L}\frac{d{\hat{\omega }}_{e}}{dt}={\hat{n}}_{p}{\hat{V}}_{s}-\frac{3}{2}{{\hat{n}}_{p}}^{2}{\hat{q}}_{r}{\hat{v}}_{q}-\hat{R}{\hat{\omega }}_{e} \end{array}\right.$$

Through the following transformations:

(28) $$\mathrm{VPMSG}:\;t=\tau_{1}\tilde{t}\;\mathrm{and}\;x=\xi_{1}\tilde{x}$$

(29) $$\mathrm{VPCSG}:\;t=\tau_{2}\tilde{t}\;\mathrm{and}\;y=\xi_{2}\tilde{x}$$

the unified model is derived as follows:

(30) $$\left\{\begin{array}{l} \frac{dz_{1}}{d\tilde{t}}=-z_{1}+z_{3}z_{2}-\Lambda_{d}\\ \frac{dz_{2}}{d\tilde{t}}=-z_{2}-z_{3}z_{1}+\gamma z_{3}-\Lambda_{q}\\ \frac{dz_{3}}{d\tilde{t}}=\Gamma_{m}-\sigma (z_{2}+z_{3}) \end{array}\right.$$

where *τ*_1_ = *L*_s_/*R*_s_, $x={\left[i_{d}\;\;i_{q}\;\;\omega_{e}\right]}^{T}$, $\tilde{x}={\left[z_{1}\;\;z_{2\;}\;z_{3}\right]}^{T}$, $k=\frac{2}{3}\frac{G}{{n_{p}}^{2}\tau_{1}\psi_{r}}$, $\xi_{1}=\left[\begin{array}{l} k\;0\;0\\ 0\;\;\;k\;0\\ 0\;\;\;0\;1/\tau_{1} \end{array}\right]$, $\gamma =\frac{\psi_{r}}{kL_{s}}$, $\Lambda_{d}=\frac{1}{R_{s}k}v_{d}$, $\Lambda_{q}=\frac{1}{R_{s}k}v_{q}$, $\Gamma_{m}=\frac{n_{p}{\tau_{1}}^{2}}{C}I_{s}$ and $\sigma =\frac{\tau_{1}G}{C}$ for VPMSG; $\tau_{2}={\hat{C}}_{s}/{\hat{G}}_{s}$, $y={\left[{\hat{v}}_{d}\;\;{\hat{v}}_{q}\;\;{\hat{\omega }}_{e}\right]}^{T}$, $\hat{k}=\frac{2}{3}\frac{\hat{R}}{{{\hat{n}}_{p}}^{2}\tau_{2}{\hat{q}}_{r}}$, $\xi_{2}=\left[\begin{array}{l} \hat{k}\;0\;0\\ 0\;\;\;\hat{k}\;0\\ 0\;\;\;0\;1/\tau_{2} \end{array}\right]$, $\gamma =\frac{{\hat{q}}_{r}}{\hat{k}{\hat{C}}_{s}}$, $\Lambda_{d}=\frac{1}{{\hat{G}}_{s}\hat{k}}{\hat{i}}_{d}$, $\Lambda_{q}=\frac{1}{{\hat{G}}_{s}\hat{k}}{\hat{i}}_{q}$, $\Gamma_{m}=\frac{{\hat{n}}_{p}{\tau_{2}}^{2}}{\hat{L}}{\hat{V}}_{s}$ and $\sigma =\frac{\tau_{2}\hat{R}}{\hat{L}}$ for VPCSG.

Furthermore, setting the time derivative in Equation (30) to zero yields the equilibrium point:

(31) $$\left(\begin{array}{l} {z_{1}}^{*}\\ {z_{2}}^{*}\\ {z_{3}}^{*} \end{array}\right)=\left(\begin{array}{c} \frac{\Gamma_{m}\xi^{*}}{\sigma }-{\xi^{*}}^{2}-\Lambda_{d}\\ \frac{\Gamma_{m}}{\sigma }-\xi^{*}\\ \xi^{*} \end{array}\right)$$

where ξ* is the solution of the following equation:

*ξ*^3^ + *a*_1_*ξ*^2^ + *a*_2_*ξ* + *a*_3_ = 0 (32)

According to the cubic formula [28], the solutions of Equation (32) can be analytically derived as follows:

(33) $$\left\{\begin{array}{l} {\xi_{1}}^{*}=T_{+}+T_{-}-\frac{1}{3}a_{1}\\ {\xi_{2,3}}^{*}=-\frac{1}{2}(T_{+}+T_{-})-\frac{1}{3}a_{1}\pm \frac{1}{2}i\sqrt{3}(T_{+}-T_{-}) \end{array}\right.$$

where $a_{1}=-\frac{\Gamma_{m}}{\sigma }$, *a*_2_ = 1 + *γ* + Λ_d_, $a_{3}=-\frac{\Gamma_{m}+\Lambda_{q}\sigma }{\sigma }$, $Q=\frac{3a_{2}-{a_{1}}^{2}}{9}$ and $R=\frac{9a_{1}a_{2}-27a_{3}-2{a_{1}}^{3}}{54}$, $T_{+}=\sqrt[{3}]{R+\sqrt{\Delta }}$, *T*_−_ = −*Q*/*T*_+_ and Δ = *Q*^3^ + *R*^2^. Therefore, three equilibria, namely *E*_1_*, *E*_2_*, and *E*_3_*, correspond to *ξ*_1_*, *ξ*_2_*, and *ξ*_3_*, respectively. When Δ ≤ 0, the unified model (30) admits at least two distinct real equilibria; otherwise, it has a unique equilibrium.

In grid-connected mode, the grid parameters are specified as follows:

Grid-Side voltages of the VPMSG: *v*_a_ = *U*_m_sin(*ω*_g_*t* + *θ*_v_), *v*_b_ = *U*_m_sin(*ω*_g_*t* + *θ*_v_ − 2/3π), and *v*_c_ = *U*_m_sin(*ω*_g_*t* + *θ*_v_ + 2/3π), where *θ*_v_, *ω*_g_, and *U*_m_ are the equilibrium phase angle, electrical angular frequency, and amplitude of the grid voltages. Here, *ω*_g_ = 2π*f*_g_, *f*_g_ denotes the grid frequency.

Grid-side currents of the VPCSG: ${\hat{i}}_{a}=I_{m}\sin(\omega_{g}t+\theta_{i})$, ${\hat{i}}_{b}=I_{m}\sin(\omega_{g}t-\frac{2}{3}\pi +\theta_{i})$ and ${\hat{i}}_{c}=I_{m}\sin(\omega_{g}t+\frac{2}{3}\pi +\theta_{i})$, where *θ*_i_ and *I*_m_ are the equilibrium phase angle and amplitude of the grid currents, respectively.

Applying the Park transformation yields

(34) $$\left\{\begin{array}{l} v_{d}=U_{m}\sin\left[\left(\omega_{g}-\omega_{e}\right)t+\theta_{v}\right]\\ v_{q}=-U_{m}\cos\left[\left(\omega_{g}-\omega_{e}\right)t+\theta_{v}\right] \end{array}\right.\;\text{for the VPMSG}$$

(35) $$\left\{\begin{array}{l} {\hat{i}}_{d}=I_{m}\sin\left[\left(\omega_{g}-{\hat{\omega }}_{e}\right)t+\theta_{i}\right]\\ {\hat{i}}_{q}=-I_{m}\cos\left[\left(\omega_{g}-{\hat{\omega }}_{e}\right)t+\theta_{i}\right] \end{array}\right.\;\text{for the VPCSG}.$$

Whether the *dq*-frame grid voltages and currents are constant or time-varying depends on the relationship between *ω*_g_ and *ω*_e_ (or *ω*_g_ and ${\hat{\omega }}_{e}$). Standalone mode is a special case of grid-connected operation where *ω*_g_ = *ω*_e_ for the VPMSG and *ω*_g_ = ${\hat{\omega }}_{e}$ for the VPCSG; consequently, *v*_d_, *v*_q_, ${\hat{i}}_{d}$, and ${\hat{i}}_{q}$ remain constant. Therefore, the stability of both the VPMSG and the VPCSG is analyzed under standalone and grid-connected modes.

### 3.1. Stability Analysis in Standalone Operation

#### 3.1.1. Λ_d_ = Λ_q_ = Γ_m_ = 0

By removing all excitation terms, Equation (30) simplifies to

(36) $$\left\{\begin{array}{l} \frac{dz_{1}}{d\tilde{t}}=-z_{1}+z_{3}z_{2}\\ \frac{dz_{2}}{d\tilde{t}}=-z_{2}-z_{3}z_{1}+\gamma z_{3}\\ \frac{dz_{3}}{d\tilde{t}}=-\sigma (z_{2}+z_{3}) \end{array}\right.$$

Setting the time derivative in Equation (36) to zero yields the equilibrium point:

(37) $$\left(\begin{array}{l} {z_{1}}^{*}\\ {z_{2}}^{*}\\ {z_{3}}^{*} \end{array}\right)=\left(\begin{array}{c} 0\\ 0\\ 0 \end{array}\right)$$

The Jacobian matrix is given by

(38) $$J_{\mathrm{ac}}=\left(\begin{array}{ccc} -1 & {z_{3}}^{*} & {z_{2}}^{*}\\ -{z_{3}}^{*} & -1 & \gamma -{z_{1}}^{*}\\ 0 & -\sigma & -\sigma \end{array}\right)$$

Solving the characteristic equation det(*λ***I** − *J*_ac_) = 0 yields the eigenvalues:

(39) $$\left\{\begin{array}{l} \lambda_{1}=-1\\ \lambda_{2,3}=\frac{-(1+\sigma )\pm \sqrt{{\left(\sigma -1\right)}^{2}-4\;\gamma \sigma }}{2} \end{array}\right.$$

The signs of the eigenvalues are analyzed as follows:

CASE 1: (*σ* − 1)^2^ − 4*γσ* ≥ 0

*λ*_2_ and *λ*_3_ are real eigenvalues. Since *λ*_2_ × *λ*_3_ = *σ*(1 + *γ*) > 0 and *λ*_2_ + *λ*_3_ = −(*σ* + 1) < 0, both *λ*_2_ and *λ*_3_ are negative real numbers.

CASE 2: (*σ* − 1)^2^ − 4*γσ* < 0

*λ*_2_ and *λ*_3_ form a complex conjugate pair with negative real parts since −(1 + *σ*)/2 < 0.

Therefore, all eigenvalues have negative real parts, implying local asymptotic stability of the equilibrium point. The representative phase trajectory of system (36) is plotted in Figure 4.

#### 3.1.2. Λ_d_ ≠ 0, Λ_q_ ≠ 0, and Γ_m_ ≠ 0

In standalone mode, it can be concluded that *ω*_g_ = *ω*_e_ for the VPMSG and *ω*_g_ = ${\hat{\omega }}_{e}$ for the VPCSG, so we can have Λ_d_ = constant and Λ_q_ = constant. For convenience of analysis, by applying the following transformations:

(40) $${\tilde{z}}_{1}=z_{1}-{z_{1}}^{*},\;{\tilde{z}}_{2}=z_{2}-{z_{2}}^{*},\;{\tilde{z}}_{3}=z_{3}-{z_{3}}^{*}$$

Equation (30) is recast into the following dynamical system:

(41) $$\dot{\tilde{z}}=\left(\begin{array}{c} {\dot{\tilde{z}}}_{1}\\ {\dot{\tilde{z}}}_{2}\\ {\dot{\tilde{z}}}_{3} \end{array}\right)=A\tilde{z}+N=\left(\begin{array}{ccc} -1 & z_{3}^{*} & z_{2}^{*}\\ -z_{3}^{*} & -1 & \gamma -z_{1}^{*}\\ 0 & -\sigma & -\sigma \end{array}\right)\left(\begin{array}{c} {\tilde{z}}_{1}\\ {\tilde{z}}_{2}\\ {\tilde{z}}_{3} \end{array}\right)+\left(\begin{array}{c} {\tilde{z}}_{2}{\tilde{z}}_{3}\\ -{\tilde{z}}_{1}{\tilde{z}}_{3}\\ 0 \end{array}\right)$$

Choose the Lyapunov function candidate as:

(42) $$V(\tilde{z})=D_{\phi }(\tilde{z},0)=\phi (\tilde{z})-\phi (0)-\langle \nabla \phi (0),\tilde{z}\rangle ={\tilde{z}}^{T}P\tilde{z}$$

with the strictly convex generator $\phi (\tilde{z})={\tilde{z}}^{T}P\tilde{z}$. Here, $\tilde{z}$ = (${\tilde{z}}_{1}$, ${\tilde{z}}_{2}$, ${\tilde{z}}_{3}$)^T^, *p* > 0, *q* > 0, *r* = *q*/*p*, and

$$P=\left(\begin{array}{ccc} p & 0 & 0\\ 0 & p & 0\\ 0 & 0 & q \end{array}\right).$$

So, the time derivative of $V(\tilde{z})$ can be derived as

(43) $$\begin{array}{l} \dot{V}(\tilde{z})={\dot{\tilde{z}}}^{T}P\tilde{z}+{\tilde{z}}^{T}P\dot{\tilde{z}}\\ =(A\tilde{z}+N{)}^{T}P\tilde{z}+{\tilde{z}}^{T}P(A\tilde{z}+N)\\ ={\tilde{z}}^{T}(A^{T}P+\mathrm{PA})\tilde{z}\\ =-{\tilde{z}}^{T}Q\tilde{z} \end{array}$$

Here,

$$Q=-(A^{T}P+\mathrm{PA})=\left(\begin{array}{ccc} 2\;p & 0 & -p\;{z_{2}}^{*}\\ 0 & 2\;p & \mathrm{q\sigma}-p\;\left(\gamma -{z_{1}}^{*}\right)\\ -p\;{z_{2}}^{*} & \mathrm{q\sigma}-p\;\left(\gamma -{z_{1}}^{*}\right) & 2\;q\;\sigma \end{array}\right).$$

The equilibrium point ${\tilde{z}}^{*}$ is locally asymptotically stable if $\dot{V}$ is negative definite, which requires all leading principal minors of **Q** to be positive. The first leading principal minor *Q*_1_ = 2*p* > 0, The second leading principal minor is *Q*_2_ = 4*p*^2^ > 0, and the third leading principal minor is

(44) $$Q_{3}=\det(Q)=2p^{3}[4\mathrm{r\sigma}-{\left(\gamma -z_{1}^{*}-\mathrm{r\sigma}\right)}^{2}-{\left(z_{2}^{*}\right)}^{2}]>0.$$

Consequently, the stability condition is established as follows:

(45) $$\Xi =4\mathrm{r\sigma}-{\left(\gamma -z_{1}^{*}-\mathrm{r\sigma}\right)}^{2}-{\left(z_{2}^{*}\right)}^{2}>0$$

It is known that $r^{*}=\arg\underset{r>0}{\max}\Xi (r)=\frac{2+(\gamma -z_{1}^{*})}{\sigma }$, so we have

(46) $$\Xi (r^{*})=4+4(\gamma -z_{1}^{*})-{\left(z_{2}^{*}\right)}^{2}$$

Therefore, when Ξ > 0, the unified system is locally asymptotically stable at the equilibrium point ${\tilde{z}}^{*}$. The representative phase trajectory of the system (30) is plotted in Figure 5.

The Lyapunov function candidate $V(\tilde{z})={\tilde{z}}^{T}P\tilde{z}$ admits a rigorous interpretation as a generalized entropy measure through the lens of Bregman divergence. So, $V(\tilde{z})$ is exactly the Bregman divergence $D_{\phi }(\tilde{z},0)$ from the equilibrium state. This places our stability analysis within the unified framework of information geometry, where Shannon entropy and quadratic Lyapunov functions are all special cases of Bregman-type functionals [29,30]. From this perspective, $\dot{V}(\tilde{z})\leq 0$ represents the monotonic decay of a generalized entropy, providing an information-geometric characterization of the damping-induced convergence in the VSG system. We emphasize that the Bregman divergence $D_{\phi }(\tilde{z},0)$ should be understood as a generalized, information-theoretic entropy measure, not as the thermodynamic entropy of the physical VSG. The thermodynamic entropy of the inverter system is governed by Joule heating in resistive components and switching losses in the semiconductor devices—processes that are not resolved by the electromechanical control-level model studied here. The irreversible process captured by ${\dot{D}}_{\phi }\leq 0$ at the macroscopic level is the damping-induced convergence to synchrony, with the virtual damping coefficient *G* or $\hat{R}$ serving as the phenomenological dissipation parameter.

### 3.2. Stability Analysis in Grid-Connected Operation

In grid-connected mode, Equation (30) can be rewritten as

(47) $$\left\{\begin{array}{l} \frac{dz_{1}}{d\tilde{t}}=-z_{1}+z_{3}z_{2}-\Theta_{m}\sin\left[\left({\tilde{\omega }}_{g}-z_{3}\right)\tilde{t}+\theta_{0}\right]\\ \frac{dz_{2}}{d\tilde{t}}=-z_{2}-z_{3}z_{1}+\gamma z_{3}+\Theta_{m}\cos\left[\left({\tilde{\omega }}_{g}-z_{3}\right)\tilde{t}+\theta_{0}\right]\\ \frac{dz_{3}}{d\tilde{t}}=\Gamma_{m}-\sigma (z_{2}+z_{3}) \end{array}\right.$$

where $\Theta_{m}=\frac{1}{R_{s}k}U_{m}$, *θ*_0_ = *θ*_v_ and ${\tilde{\omega }}_{g}=\omega_{g}\tau_{1}$ for the VPMSG; $\Theta_{m}=\frac{1}{{\hat{G}}_{s}\hat{k}}I_{m}$, *θ*_0_ = *θ*_i_ and ${\tilde{\omega }}_{g}=\omega_{g}\tau_{2}$ for the VPCSG. Equation (47) constitutes a complete nonlinear dynamic model capable of capturing the full dynamics of the system, including non-synchronous transients, where ${\tilde{\omega }}_{g}\neq z_{3}$. The term $\left({\tilde{\omega }}_{g}-z_{3}\right)$ explicitly captures the instantaneous slip frequency, and no prior synchronization assumption is embedded within the equation itself. For grid-connected VSGs, maintaining synchronization is a fundamental operational prerequisite. Accordingly, the stability analysis of the frequency-locked state is presented below.

In steady state, the angular frequency in Equation (47) is synchronized with the grid angular frequency; thus, ${\tilde{\omega }}_{g}=z_{3}$. Therefore, the following conditions are satisfied:

(48) $$\phi (\tilde{t})=\left({\tilde{\omega }}_{g}-z_{3}\right)\tilde{t}+\theta_{0},\;{\dot{z}}_{3}=0\;\mathrm{and}\;\dot{\phi }(\tilde{t})=0$$

Substitution of ${\dot{z}}_{3}=0$ into $0=\Gamma_{m}-\sigma (z_{2}+{\tilde{\omega }}_{g})$ yields

(49) $${{\tilde{z}}_{2}}^{*}=\frac{\Gamma_{m}}{\sigma }-{\tilde{\omega }}_{g}$$

Furthermore, by setting $\frac{dz_{1}}{d\tilde{t}}=0$ and $\frac{dz_{2}}{d\tilde{t}}=0$, it is derived that

(50) $$\left\{\begin{array}{l} 0=-z_{1}+{\tilde{\omega }}_{g}{{\tilde{z}}_{2}}^{*}-\Theta_{m}\sin\theta_{0}\\ 0=-{{\tilde{z}}_{2}}^{*}-{\tilde{\omega }}_{g}z_{1}+\gamma {\tilde{\omega }}_{g}+\Theta_{m}\cos\theta_{0} \end{array}\right.$$

So,

(51) $$\left\{\begin{array}{l} z_{1}={\tilde{\omega }}_{g}{{\tilde{z}}_{2}}^{*}-\Theta_{m}\sin\theta_{0}\\ {{\tilde{z}}_{2}}^{*}+{\tilde{\omega }}_{g}z_{1}-\gamma {\tilde{\omega }}_{g}=\Theta_{m}\cos\theta_{0} \end{array}\right.$$

Substituting the first expression in Equation (51) into the second yields

(52) $$\frac{{{\tilde{z}}_{2}}^{*}(1+{{\tilde{\omega }}_{g}}^{2})-\gamma {\tilde{\omega }}_{g}}{\Theta_{m}\sqrt{1+{{\tilde{\omega }}_{g}}^{2}}}=\sin(\theta_{0}+\arctan(\frac{1}{{\tilde{\omega }}_{g}}))$$

From Equation (52), the equilibrium phase angle *θ*_0_ admits two analytical solutions:

(53) $$\left\{\begin{array}{l} {\theta_{0}}^{\left(1\right)}=-\arctan(\frac{1}{{\tilde{\omega }}_{g}})+\arcsin\frac{{{\tilde{z}}_{2}}^{*}(1+{{\tilde{\omega }}_{g}}^{2})-\gamma {\tilde{\omega }}_{g}}{\Theta_{m}\sqrt{1+{{\tilde{\omega }}_{g}}^{2}}}\\ {\theta_{0}}^{\left(2\right)}=-\arctan(\frac{1}{{\tilde{\omega }}_{g}})+\pi -\arcsin\frac{{{\tilde{z}}_{2}}^{*}(1+{{\tilde{\omega }}_{g}}^{2})-\gamma {\tilde{\omega }}_{g}}{\Theta_{m}\sqrt{1+{{\tilde{\omega }}_{g}}^{2}}} \end{array}\right.$$

Substituting Equation (49) into Equation (52) yields

(54) $$\Pi =\left|\frac{(\frac{\Gamma_{m}}{\sigma }-{\tilde{\omega }}_{g})(1+{{\tilde{\omega }}_{g}}^{2})-\gamma {\tilde{\omega }}_{g}}{\Theta_{m}\sqrt{1+{{\tilde{\omega }}_{g}}^{2}}}\right|\leq 1$$

Equation (54) gives the necessary condition for the angular frequency of the unified model to be locked to the grid angular frequency, as illustrated for the VPMSG and the VPCSG in Figure 6. It can be seen that the regions corresponding to ${\tilde{\omega }}_{g}=z_{3}$ should appear among the regions with Π ≤ 1. However, when Π ≤ 1, the stability of the unified model requires further discussion, as explained below.

In the frequency-locked state, the equilibrium point of the grid-connected unified system (47) can be rewritten as

(55) $$\left\{\begin{array}{l} {{\tilde{z}}_{1}}^{*}={\tilde{\omega }}_{g}{{\tilde{z}}_{2}}^{*}-\Theta_{m}\sin\theta_{0}\\ {{\tilde{z}}_{2}}^{*}=\frac{\Gamma_{m}}{\sigma }-{\tilde{\omega }}_{g}\\ {{\tilde{z}}_{3}}^{*}={\tilde{\omega }}_{g} \end{array}\right.$$

Here, there are two equilibrium points *E*_1_* and *E*_2_* corresponding to *θ*_0_^(1)^ and *θ*_0_^(2)^.

Therefore, the Jacobian matrix can be obtained as

(56) $${\tilde{J}}_{\mathrm{ac}}=\left(\begin{array}{ccc} -1 & {\tilde{\omega }}_{g} & {{\tilde{z}}_{2}}^{*}\\ -{\tilde{\omega }}_{g} & -1 & \gamma -{{\tilde{z}}_{1}}^{*}\\ 0 & -\sigma & -\sigma \end{array}\right)$$

The characteristic equation $\det(\lambda I-{\tilde{J}}_{\mathrm{ac}})=0$ is derived as

*λ*^3^ + *b*_1_*λ*^2^ + *b*_2_*λ* + *b*_3_ = 0 (57)

Here, *b*_1_ = *σ* + 2, $b_{2}={{\tilde{\omega }}_{g}}^{2}+2\;\sigma +\sigma (\gamma \;-{{\tilde{z}}_{1}}^{*})+1$, $b_{3}=\sigma \;({{\tilde{\omega }}_{g}}^{2}-\;{{\tilde{z}}_{2}}^{*}{\tilde{\omega }}_{g}+1+\gamma \;-{{\tilde{z}}_{1}}^{*})$.

Applying the cubic formula, the eigenvalues are analytically obtained as follows:

(58) $$\left\{\begin{array}{l} \lambda_{1}={\hat{T}}_{+}+{\hat{T}}_{-}-\frac{1}{3}b_{1}\\ \lambda_{2,3}=-\frac{1}{2}({\hat{T}}_{+}+{\hat{T}}_{-})-\frac{1}{3}b_{1}\pm \frac{1}{2}i\sqrt{3}({\hat{T}}_{+}-{\hat{T}}_{-}) \end{array}\right.$$

where $\hat{Q}=\frac{3b_{2}-{b_{1}}^{2}}{9}$ and $\hat{R}=\frac{9b_{1}b_{2}-27b_{3}-2{b_{1}}^{3}}{54}$, ${\hat{T}}_{+}=\sqrt[{3}]{\hat{R}+\sqrt{\hat{\Delta }}}$, ${\hat{T}}_{-}=-\hat{Q}/{\hat{T}}_{+}$ and $\hat{\Delta }={\hat{Q}}^{3}+{\hat{R}}^{2}$. Providing that all eigenvalues possess negative real parts, the system is asymptotically stable.

In the stable frequency-locked state, the typical phase trajectory of the unified model (47) is shown in Figure 7. The system is stabilized at the equilibrium point *E*_2_*. The state variable *z*_3_ converges to the grid angular frequency ${\tilde{\omega }}_{g}$, as shown in Figure 8.

## 4. Numerical Simulations

Without loss of generality, the operating parameters of the unified model are chosen as follows: *L*_s_ = 2 mH, *R*_s_ = 200 mΩ, *C* = 470 μF, and *G* = 0.1 mS for the VPMSG; ${\hat{C}}_{s}=100\;\mu F$, ${\hat{G}}_{s}=10\;\mathrm{mS}$, $\hat{L}=2\;\mathrm{mH}$, and $\hat{R}=15\;m\Omega$ for the VPCSG. The SPWM modulation technique is employed herein, and the modulation constraints must be satisfied as follows: (i) $\sqrt{{v_{d}}^{2}+{v_{q}}^{2}}\leq \frac{1}{2}v_{\mathrm{dc}}$ for VPMSG; (ii) $\sqrt{{{\hat{i}}_{d}}^{2}+{{\hat{i}}_{q}}^{2}}\leq \frac{1}{2}{\hat{i}}_{L}$ for VPCSG. To verify the stability of the unified model, the system is analyzed in standalone mode and grid-connected mode, respectively.

### 4.1. Standalone Mode

Based on Equation (46), Ξ is calculated as 1.5 × 10^4^ > 0 for the VPMSG and 460.1 > 0 for the VPCSG, so they are stable. The typical waveforms are shown in Figure 9. As observed, the output three-phase currents and voltages are symmetrical, and the output angular frequency is stabilized at *f*_g_ = 50 Hz for both generators. The swing equations of the VPMSG and VPCSG, derived from Kirchhoff’s laws, can be reformulated as energy conservation equations:

(59) $$\mathrm{VPMSG}:\;Cv_{\mathrm{dc}}\frac{dv_{\mathrm{dc}}}{dt}=I_{s}v_{\mathrm{dc}}-i_{\mathrm{dc}}v_{\mathrm{dc}}-G{v_{\mathrm{dc}}}^{2}$$

(60) $$\mathrm{VPCSG}:\;\hat{L}{\hat{i}}_{L}\frac{d{\hat{i}}_{L}}{dt}={\hat{V}}_{s}{\hat{i}}_{L}-{\hat{v}}_{\mathrm{dc}}{\hat{i}}_{L}-\hat{R}{{\hat{i}}_{L}}^{2}$$

Therefore, instantaneous power balance is inherently maintained. The left-hand side of Equation (59) represents the power *P*_C_ absorbed by the capacitor, while the three terms on the right-hand side correspond to the power *P*_S_ supplied by the source, the power *P*_L_ consumed by the ac-side load, and the power *P*_G_ dissipated in the conductance, respectively. Similarly, the left-hand side of Equation (60) represents the power ${\hat{P}}_{I}$ absorbed by the inductor, while the three terms on the right-hand side correspond to the power ${\hat{P}}_{S}$ supplied by the source, the power ${\hat{P}}_{L}$ consumed by the ac-side load, and the power ${\hat{P}}_{R}$ dissipated in the resistance, respectively. Figure 10 presents the corresponding simulation results.

### 4.2. Grid-Connected Mode

Under control strategies (24) and (25), the VSI and the CSI function as the VPMSG and the VPCSG, respectively. When the unified model (47) falls within the frequency-locked regions shown in Figure 6, its stability must be further verified through eigenvalue analysis to ensure that the VSG outputs stable three-phase currents or voltages. According to Equation (58), the real part of the rightmost eigenvalue *λ*_max_ is numerically computed as a function of *U*_m_ and *I*_m_, as illustrated in Figure 11. Since no additional grid resistance is incorporated into the unified model, the grid-connected system is analyzed under ideal strong-grid conditions. Notably, under strong-grid conditions, the VPCSG exhibits a wider synchronous stability region than the VPMSG despite grid parameter variations when the control parameters are fixed, as shown in Figure 11. When Π < 1 and Re(*λ*_max_) < 0, the system is stabilized at the grid frequency *f*_g_ = 50 Hz. The typical waveforms are illustrated in Figure 12. Obviously, the electrical angular frequency *ω*_e_ can maintain synchronization with the grid frequency across a broad operating range of *U*_m_ and *I*_m._ The dynamic responses of the VPMSG under a step change in *n*_p_ from 1 rad/(s·V) to 1.3 rad/(s·V) are depicted in Figure 13a. Similarly, Figure 13b illustrates the VPCSG responses when ${\hat{n}}_{p}$ steps from 5 rad/(s·A) to 5.3 rad/(s·A). The VSGs exhibit prolonged transient periods due to open-loop operation. The ac-side active power of the VSGs exhibits a step change in response to variations in the virtual number of pole pairs.

## 5. Conclusions

The VSI and CSI are dual topologies that share a similar mathematical model. Under specific control conditions, the VSI is mathematically equivalent to a VPMSG; likewise, the CSI is mathematically equivalent to a VPCSG. Therefore, the VPMSG and VPCSG form a pair of dual VSGs that share an analogous mathematical model. A unified dimensionless model can be established to characterize their dynamic behaviors. By selecting an appropriate Lyapunov function candidate, the stability conditions for the unified system in standalone mode are analytically derived. Through eigenvalue analysis, the stability conditions for the unified model in grid-connected mode are also derived. Nevertheless, grid-connected operation necessitates that the VSGs remain locked to the grid frequency. Both frequency synchronization and stability conditions must be simultaneously satisfied to enable grid connection of the VSGs. The derived stability condition assumes linear operation. When the converter hits modulation limits (voltage saturation in VSI or current saturation in CSI), the control law is effectively overridden by the physical limits, and the linearized analysis no longer applies. It is worth noting a profound connection with entropy: the Lyapunov function *V*(*x*) is a Bregman divergence and serves as an information-theoretic generalized entropy. The condition $\dot{V}<0$ implies continuous dissipation of this generalized entropy toward the equilibrium (*V* = 0), and the frequency-locking condition constitutes the dissipativity bound for maintaining self-organized synchrony. Crucially, this macroscopic generalized entropy is distinct from the physical thermodynamic entropy of the VSG, which is strictly non-decreasing.

## Figures and Tables

**Figure 1:**
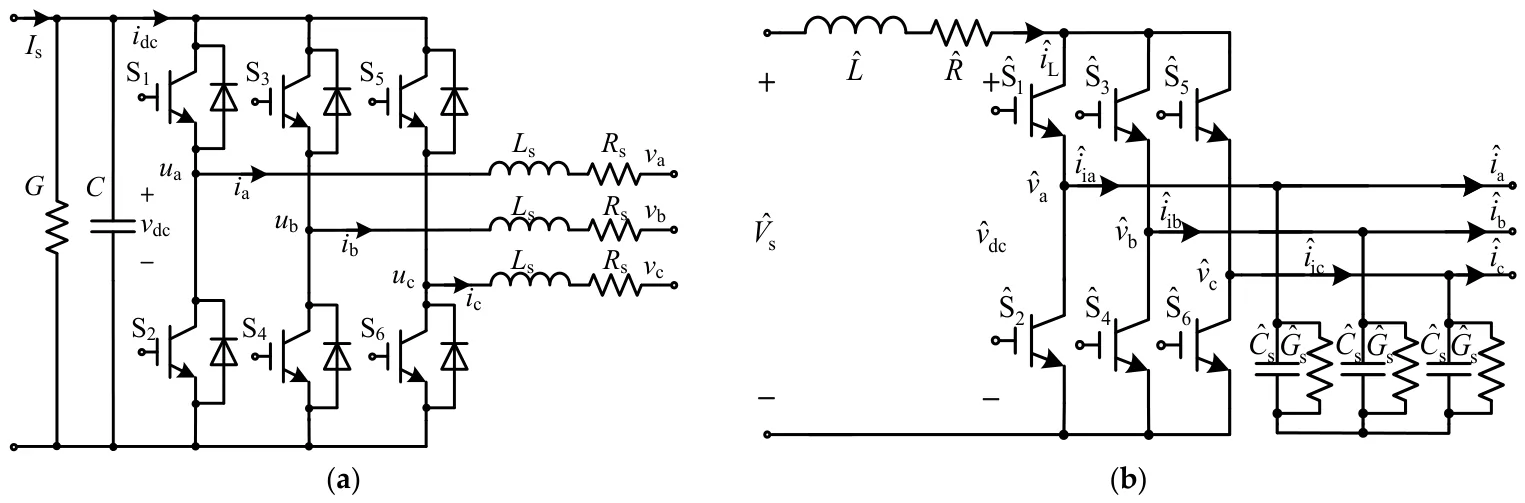
Topologies of the VSGs: (**a**) VSI. (**b**) CSI.

**Figure 2:**
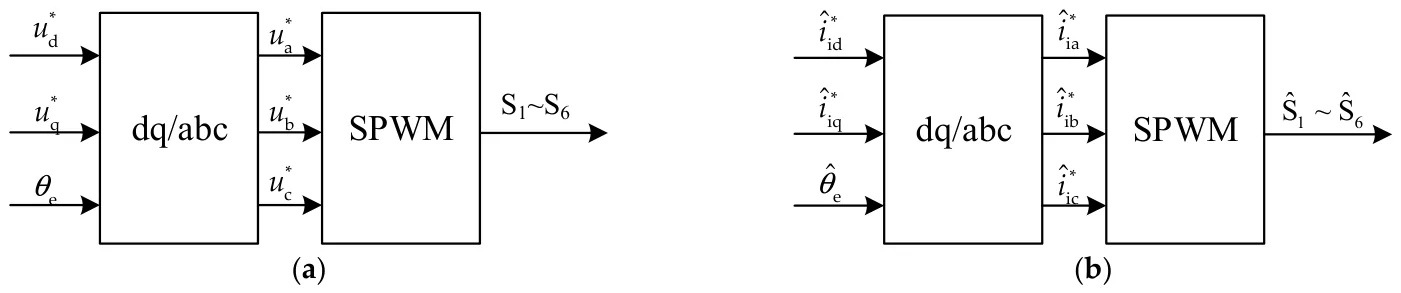
Control strategies for the VSI and CSI: (**a**) VPMSG. (**b**) VPCSG.

**Figure 3:**
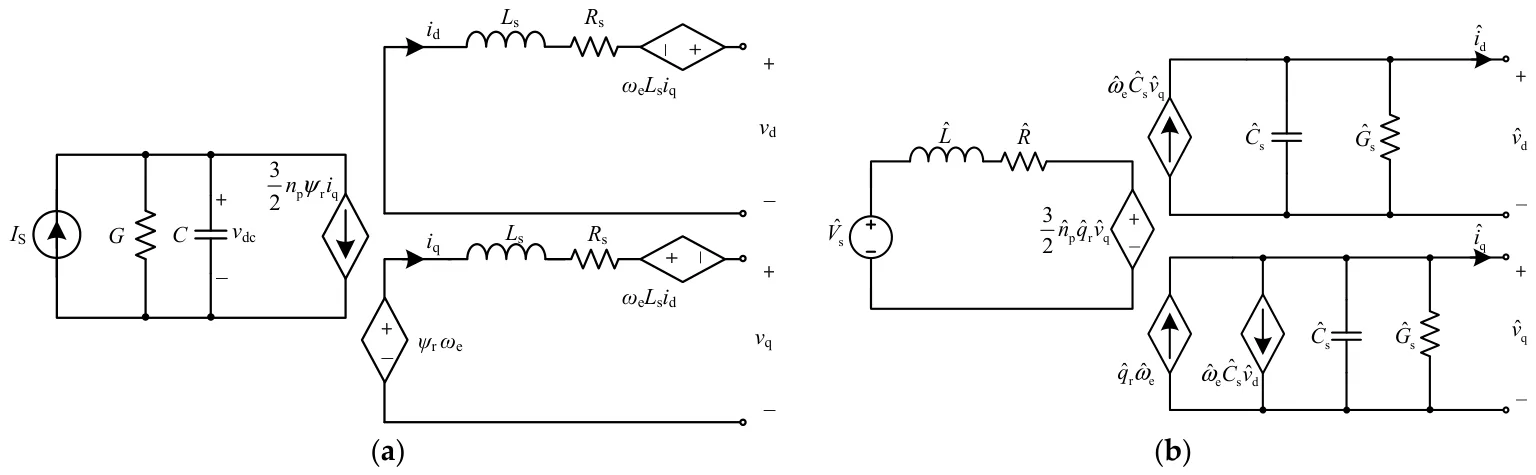
Circuit equivalent models: (**a**) VPMSG. (**b**) VPCSG.

**Figure 4:**
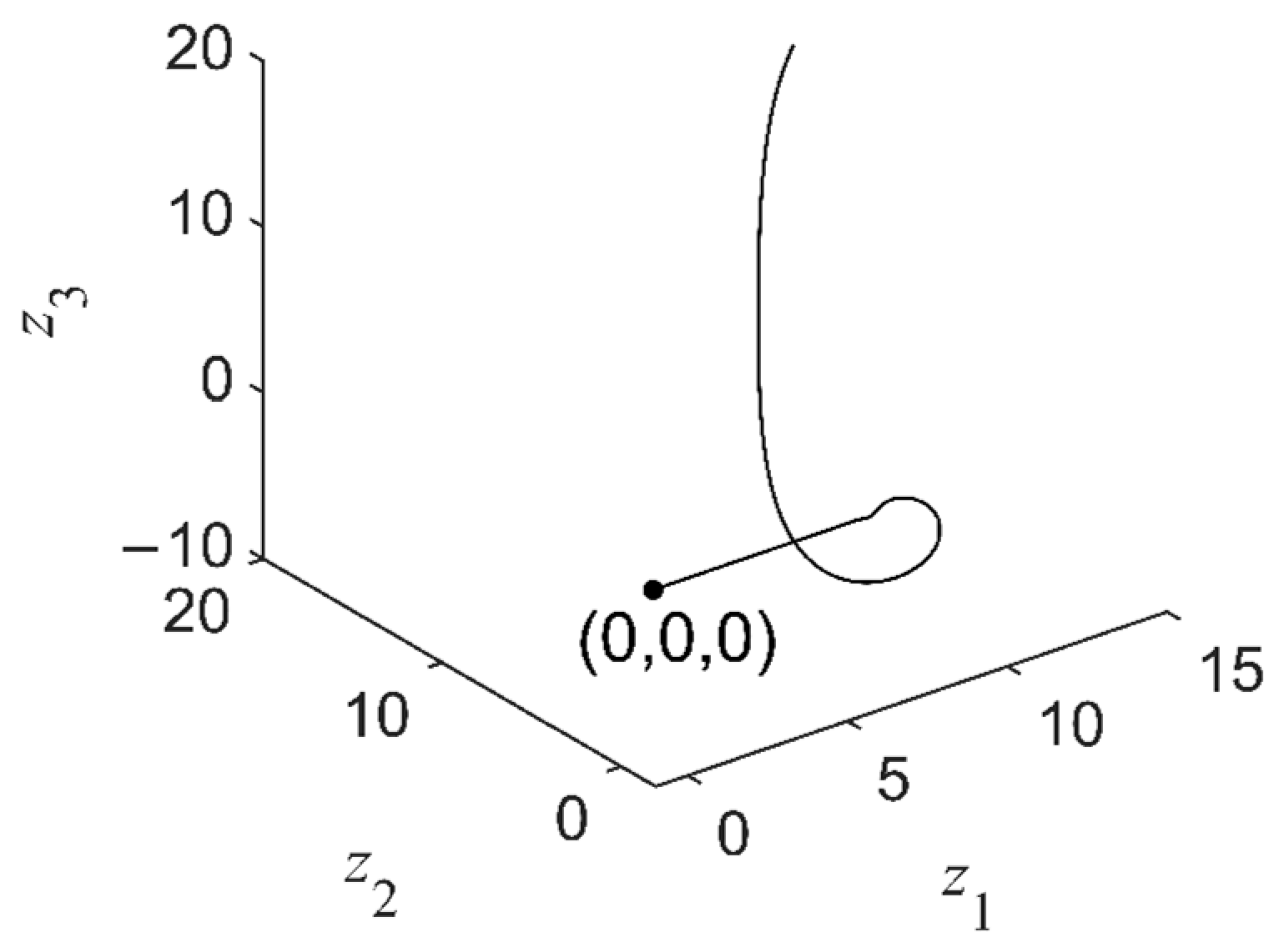
Phase trajectory of the unified model with *γ* = 44.76 and *σ* = 44.68.

**Figure 5:**
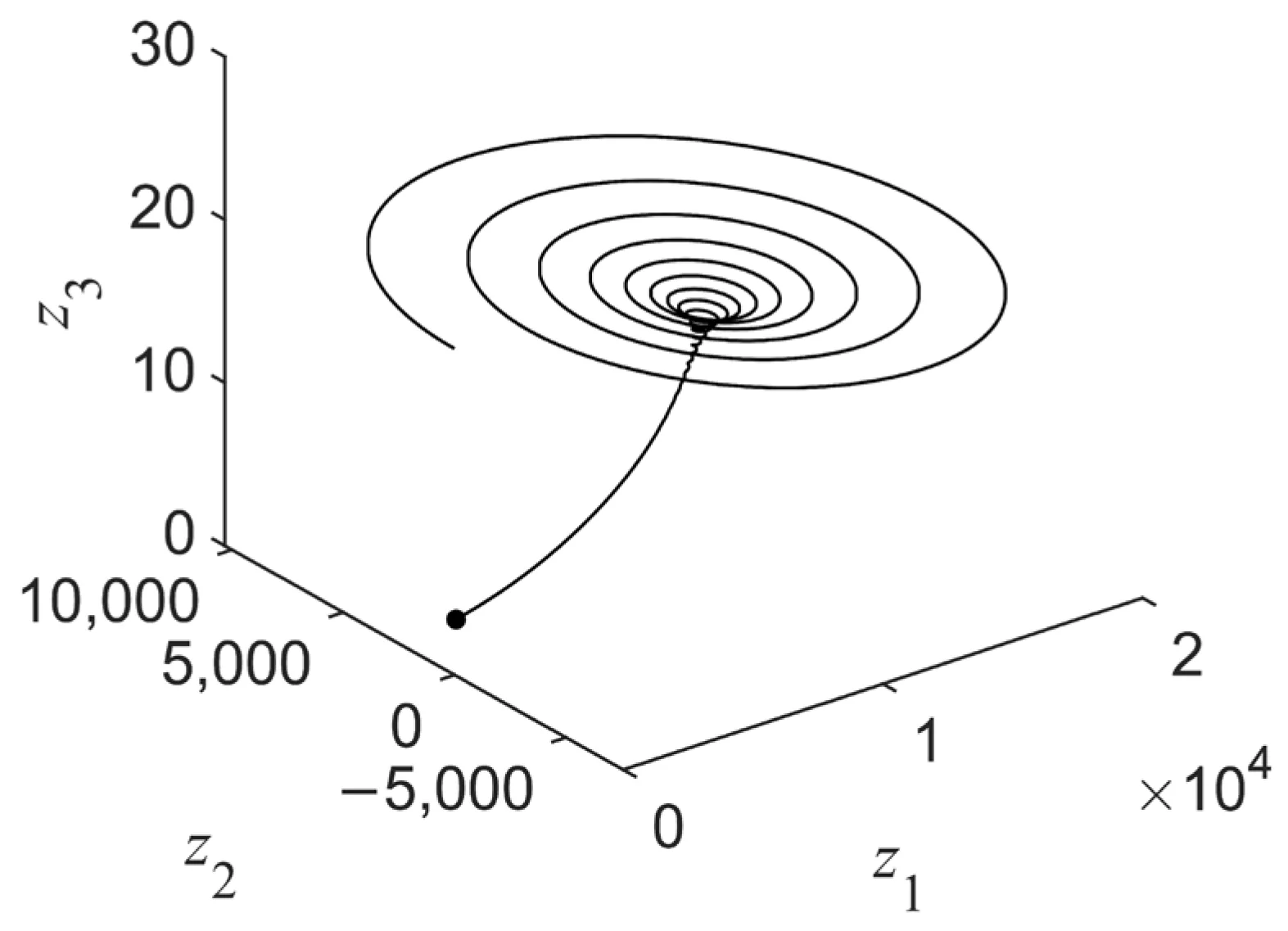
Phase trajectory of the unified model with *γ* = 1.26 × 10^4^, *σ* = 0.0021, Λ_d_ = 369, Λ_q_ = 3.9 × 10^4^, and Γ_m_ = 0.4043.

**Figure 6:**
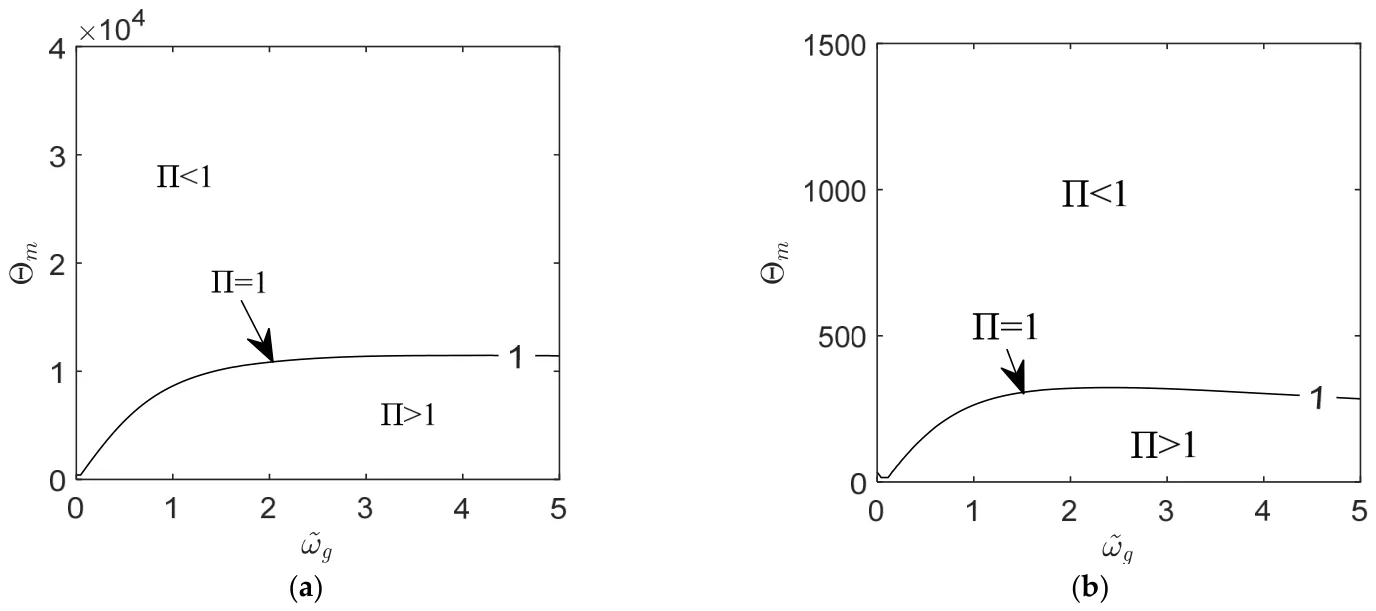
Frequency-locked regions of the unified model: (**a**) VPMSG with *γ* = 1.26 × 10^4^, *σ* = 0.0021, Θ_m_ = 3.54 × 10^4^, Γ_m_ = 0.4043, ${\tilde{\omega }}_{g}=3.14$ and *θ*_0_ = *θ*_0_^(2)^ = 3.16. (**b**) VPCSG with *γ* = 436.81, *σ* = 0.075, Θ_m_ = 1 × 10^3^, Γ_m_ = 2.5, ${\tilde{\omega }}_{g}=3.14$ and *θ*_0_ = *θ*_0_^(2)^ = 3.14.

**Figure 7:**
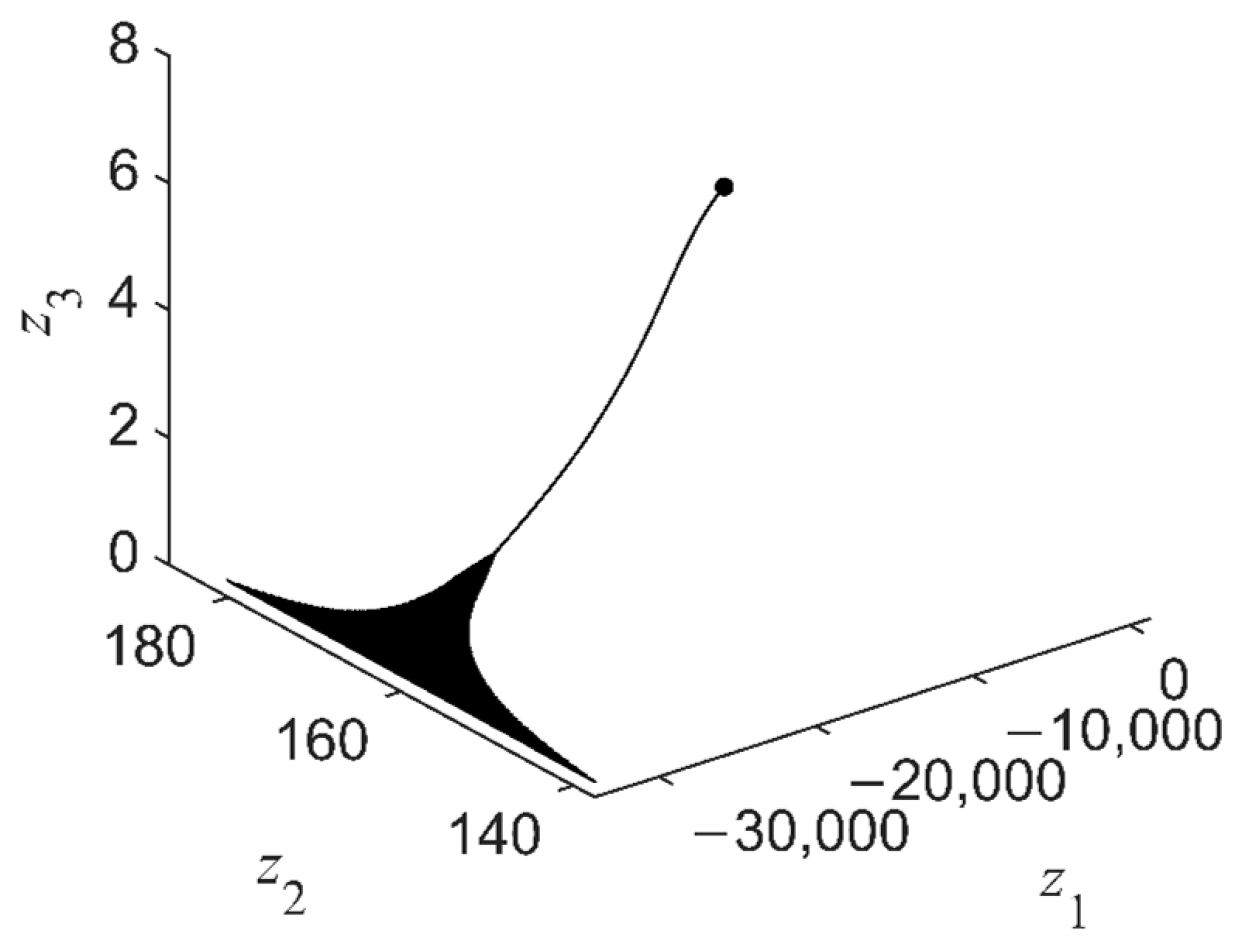
Phase trajectory of the unified model with *γ* = 1.26 × 10^4^, *σ* = 0.0021, Θ_m_ = 3.54 × 10^4^, Γ_m_ = 0.4043, ${\tilde{\omega }}_{g}=3.14$ and *θ*_0_ = *θ*_0_^(2)^ = 3.16.

**Figure 8:**
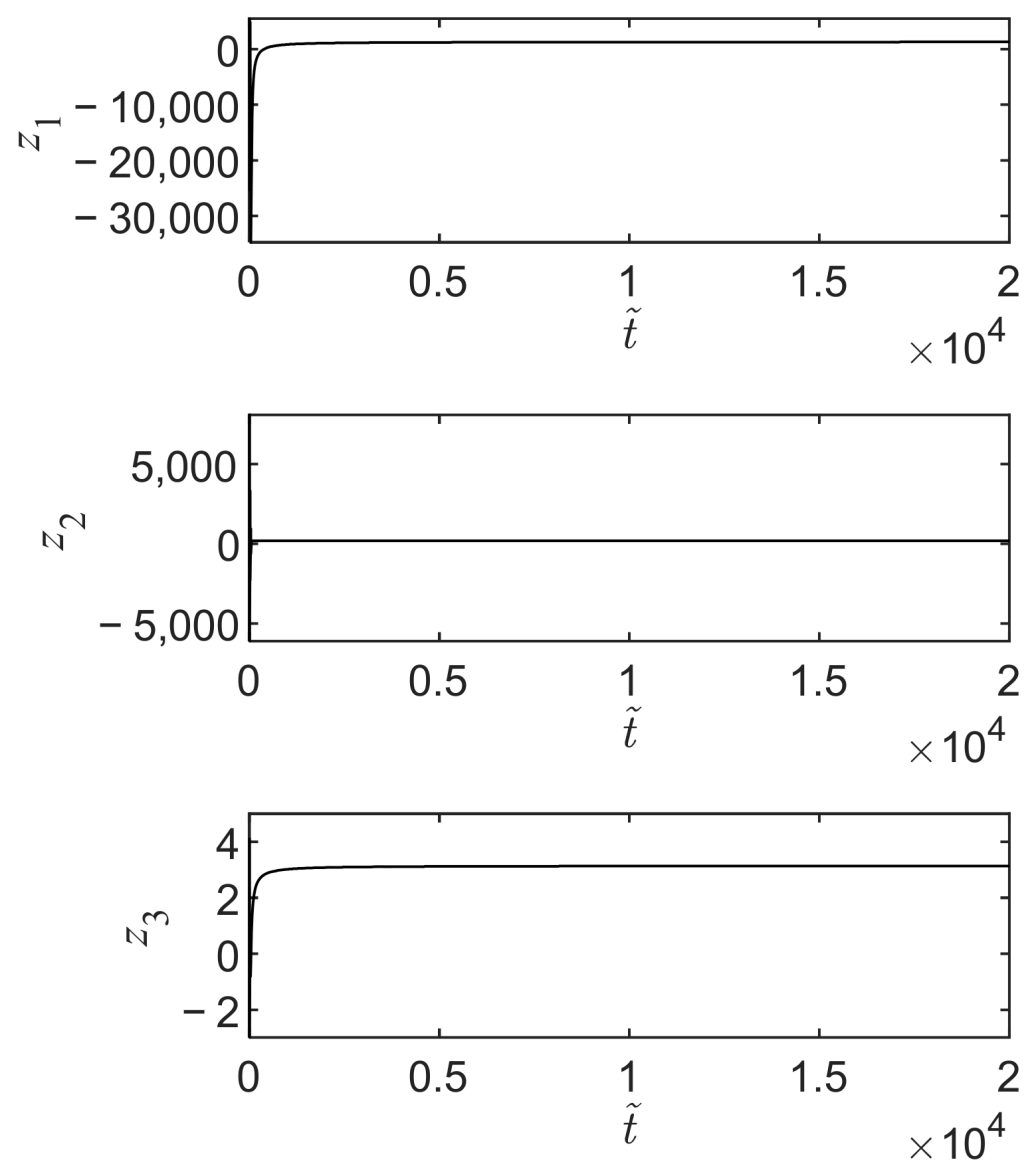
Waveforms of the grid-connected unified model with *γ* = 1.26 × 10^4^, *σ* = 0.0021, Θ_m_ = 3.54 × 10^4^, Γ_m_ = 0.4043, ${\tilde{\omega }}_{g}=3.14$ and *θ*_0_ = *θ*_0_^(2)^ = 3.16.

**Figure 9:**
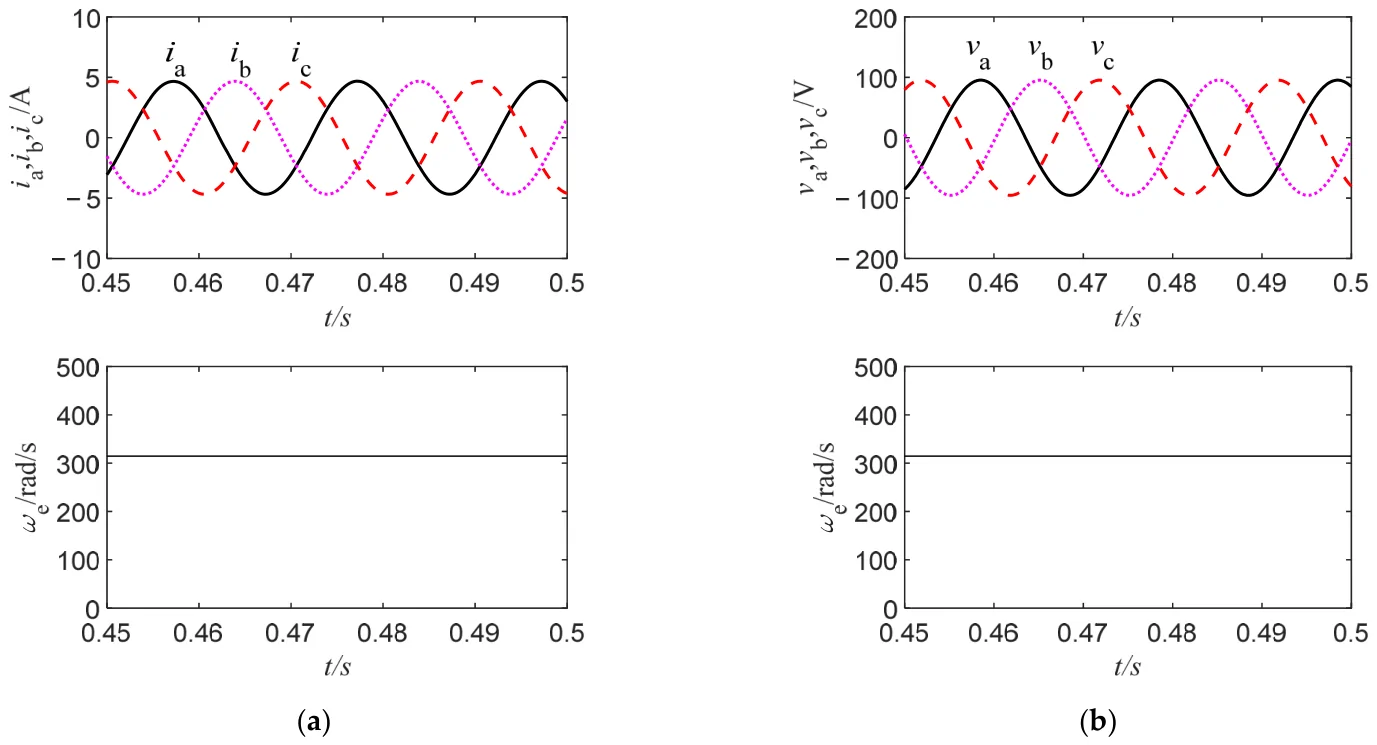
Waveforms of the unified model: (**a**) VPMSG with *I*_s_ = 1.9 A, *n*_p_ = 1 rad/(s·V), *ψ*_r_ = 0.41 Wb, *v*_d_ = 1.2 V, and *v*_q_ = 126.1 V. (**b**) VPCSG with ${\hat{V}}_{s}=10\;V$, ${\hat{n}}_{p}$ = 5 rad/(s·A), ${\hat{q}}_{r}=0.0418\;C$, ${\hat{i}}_{d}=0\;A$, and ${\hat{i}}_{q}=10\;A$.

**Figure 10:**
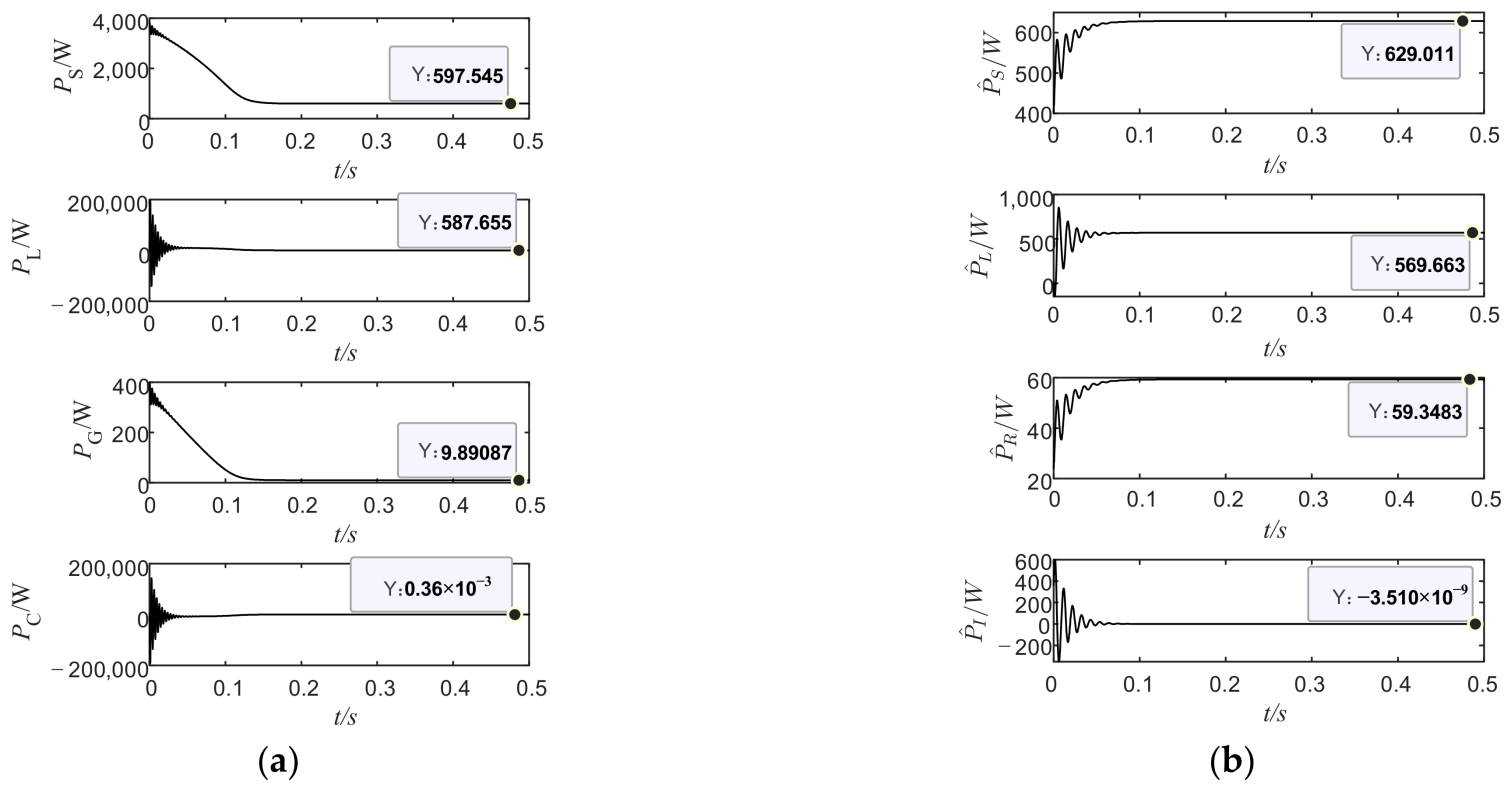
Instantaneous powers of the unified model: (**a**) VPMSG with *I*_s_ = 1.9 A, *n*_p_ = 1 rad/(s·V), *ψ*_r_ = 0.41 Wb, *v*_d_ = 1.2 V, and *v*_q_ = 126.1 V. (**b**) VPCSG with ${\hat{V}}_{s}=10\;V$, ${\hat{n}}_{p}$ = 5 rad/(s·A), ${\hat{q}}_{r}=0.0418\;C$, ${\hat{i}}_{d}=0\;A$, and ${\hat{i}}_{q}=10\;A$.

**Figure 11:**
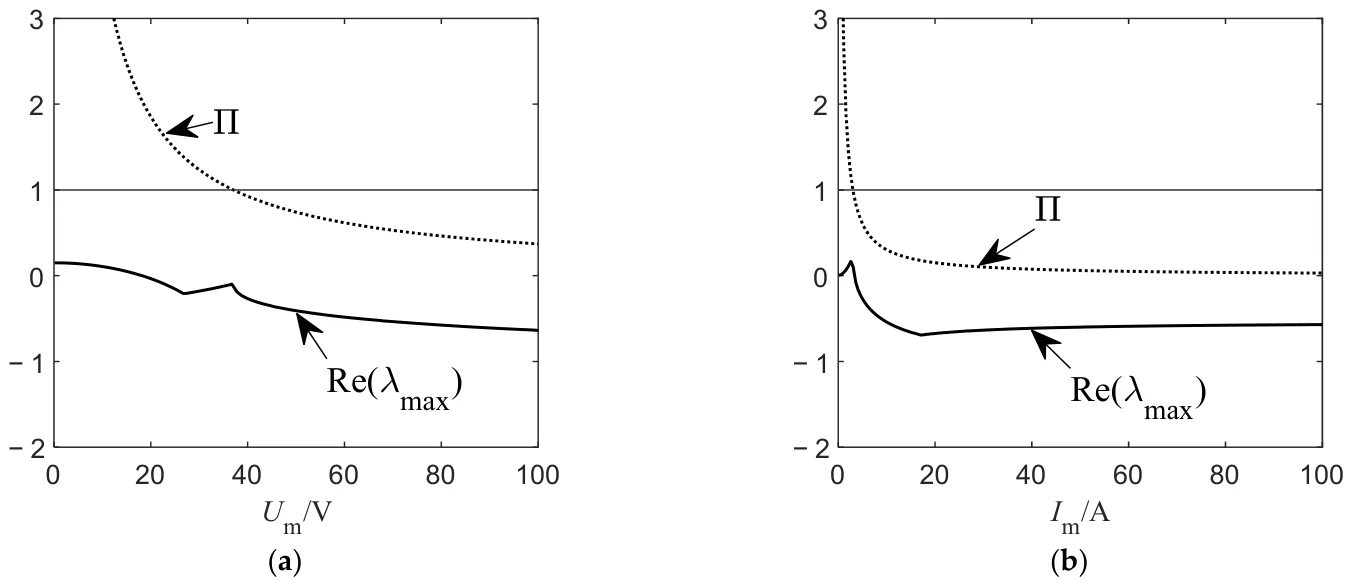
Curves of the real parts of the rightmost eigenvalues: (**a**) VPMSG. (**b**) VPCSG.

**Figure 12:**
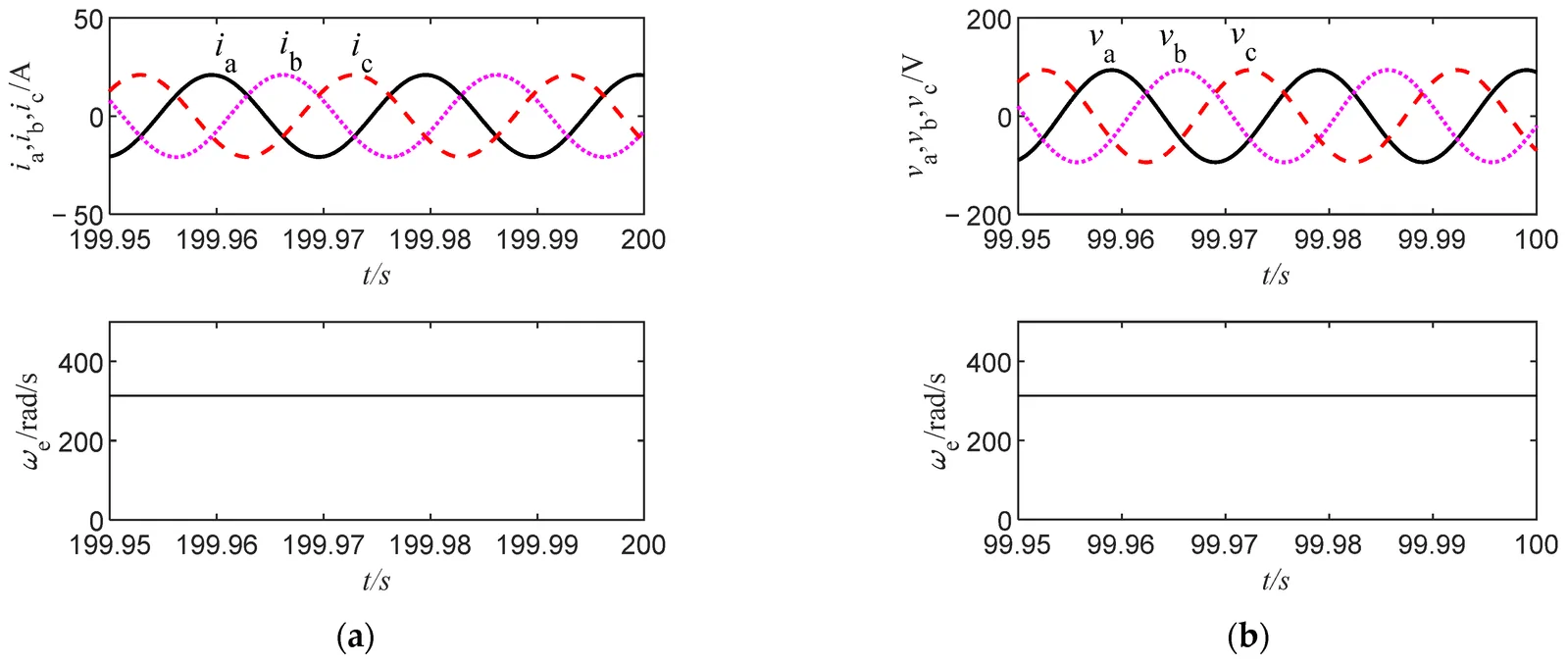
Waveforms of the unified model: (**a**) VPMSG with *I*_s_ = 1.9 A, *n*_p_ = 1 rad/(s·V), *ψ*_r_ = 0.41 Wb, *U*_m_ = 115 V, *f*_g_ = 50 Hz, and *θ*_0_ = *θ*_0_^(2)^ = 3.16. (**b**) VPCSG with ${\hat{V}}_{s}=10\;V$, ${\hat{n}}_{p}$ = 5 rad/(s·A), ${\hat{q}}_{r}=0.0418\;C$, *I*_m_ = 10 A, *f*_g_ = 50 Hz, and *θ*_0_ = *θ*_0_^(2)^ = 3.14.

**Figure 13:**
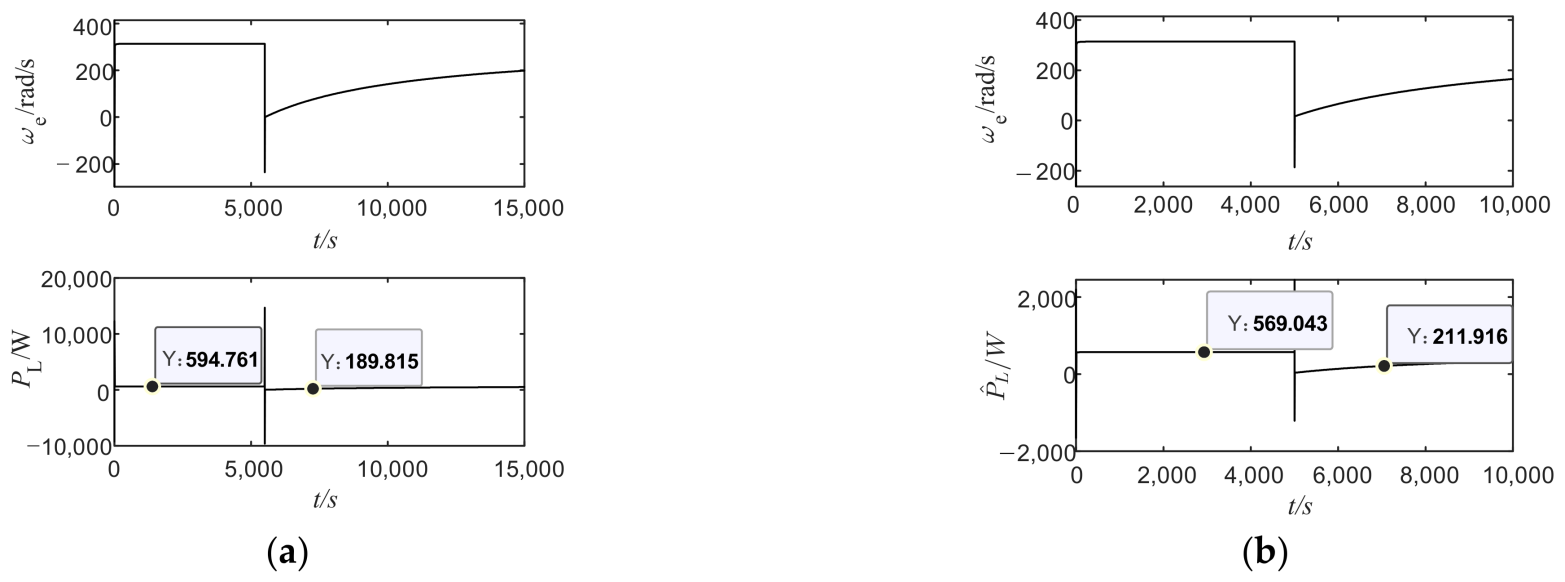
Step responses of the unified model: (**a**) VPMSG. (**b**) VPCSG.

**Table 1:** Dual terms between Equations (1) and (5).

Equation (1)		Equation (5)	
Description	Symbol	Description	Symbol
Voltage	${\vec{v}}_{s}$	Current	${\vec{\hat{i}}}_{s}$
Current	${\vec{i}}_{s}$	Voltage	${\vec{\hat{v}}}_{s}$
Flux	${\vec{\varphi }}_{s}$	Charge	${\vec{\hat{q}}}_{s}$
Resistance	*R*_s_	Conductance	${\hat{G}}_{s}$

**Table 2:** Duality between VPMSG and VPCSG.

VPMSG		VPCSG	
Description	Expression	Description	Expression
Voltage Equations	$\left\{\begin{array}{l} L_{s}\frac{di_{d}}{dt}=-v_{d}-R_{s}i_{d}+\omega_{e}L_{s}i_{q}\\ L_{s}\frac{di_{q}}{dt}=-v_{q}-R_{s}i_{q}-\omega_{e}L_{s}i_{d}+\psi_{r}\omega_{e} \end{array}\right.$	Current Equations	$\left\{\begin{array}{l} {\hat{C}}_{s}\frac{d{\hat{v}}_{d}}{dt}=-{\hat{i}}_{d}-{\hat{G}}_{s}{\hat{v}}_{d}+{\hat{\omega }}_{e}{\hat{C}}_{s}{\hat{v}}_{q}\\ {\hat{C}}_{s}\frac{d{\hat{v}}_{q}}{dt}=-{\hat{i}}_{q}-{\hat{G}}_{s}{\hat{v}}_{q}-{\hat{\omega }}_{e}{\hat{C}}_{s}{\hat{v}}_{d}+{\hat{q}}_{r}{\hat{\omega }}_{e} \end{array}\right.$
Swing Equation	$J\frac{d\omega_{e}}{dt}=n_{p}T_{m}-n_{p}T_{e}-B_{f}\omega_{e}$	Swing Equation	$J\frac{d{\hat{\omega }}_{e}}{dt}={\hat{n}}_{p}T_{m}-{\hat{n}}_{p}{\hat{T}}_{e}-B_{f}{\hat{\omega }}_{e}$
Flux Equations	$\left\{\begin{array}{l} \varphi_{d}=-L_{s}i_{d}+\psi_{r}\\ \varphi_{q}=-L_{s}i_{q} \end{array}\right.$	Charge Equations	$\left\{\begin{array}{l} {\hat{q}}_{d}=-{\hat{C}}_{s}{\hat{v}}_{d}+{\hat{q}}_{r}\\ {\hat{q}}_{q}=-{\hat{C}}_{s}{\hat{v}}_{q} \end{array}\right.$
Torque	$T_{e}=\frac{3}{2}n_{p}(\psi_{d}i_{q}-\psi_{q}i_{d})$	Torque	${\hat{T}}_{e}=\frac{3}{2}{\hat{n}}_{p}({\hat{q}}_{d}{\hat{v}}_{q}-{\hat{q}}_{q}{\hat{v}}_{d})$
Electrical Angular Frequency	*ω*_e_ = *n*_p_*ω*_m_	Electrical Angular Frequency	${\hat{\omega }}_{e}={\hat{n}}_{p}{\hat{\omega }}_{m}$
Electrical angle	*θ*_e_	Electrical angle	${\hat{\theta }}_{e}$

**Table 3:** Comparison of VPMSG and VPCSG.

Name	VPMSG	VPCSG
Swing Equations	$C\frac{dv_{\mathrm{dc}}}{dt}=I_{s}-i_{\mathrm{dc}}-Gv_{\mathrm{dc}}$	$\hat{L}\frac{d{\hat{i}}_{L}}{dt}={\hat{V}}_{s}-{\hat{v}}_{\mathrm{dc}}-\hat{R}{\hat{i}}_{L}$
Energy Storage Elements	Dc-link capacitor *C*	Dc-link inductor $\hat{L}$
Electrical Angular Frequency	*ω*_e_ = *n*_p_*v*_dc_	${\hat{\omega }}_{e}={\hat{n}}_{p}{\hat{i}}_{L}$
Virtual Input Torque	Current source *I*_s_	Voltage source ${\hat{V}}_{s}$
Virtual Electromagnetic Torque	$T_{e}=i_{\mathrm{dc}}=\frac{3}{2}n_{p}\psi_{r}i_{q}$	${\hat{T}}_{e}={\hat{v}}_{\mathrm{dc}}=\frac{3}{2}{\hat{n}}_{p}{\hat{q}}_{r}{\hat{v}}_{q}$

## Data Availability

The original contributions presented in the study are included in the article; further inquiries can be directed to the corresponding author.

## Abbreviations

The following abbreviations are used in this manuscript:

VPMSG	Virtual permanent-magnet synchronous generator
VPCSG	Virtual permanent-capacitor synchronous generator
VSG	Virtual synchronous generator
VSI	Voltage-source inverter
CSI	Current-source inverter
